# Aging: a portrait from gene expression profile in blood cells

**DOI:** 10.18632/aging.101016

**Published:** 2016-08-19

**Authors:** Elisa Calabria, Emilia Maria Cristina Mazza, Kenneth Allen Dyar, Silvia Pogliaghi, Paolo Bruseghini, Carlo Morandi, Gian Luca Salvagno, Matteo Gelati, Gian Cesare Guidi, Silvio Bicciato, Stefano Schiaffino, Federico Schena, Carlo Capelli

**Affiliations:** ^1^ Department of Neurosciences, Biomedicine and Movement Sciences, University of Verona, Verona, Italy; ^2^ Department Life Sciences, University of Modena and Reggio Emilia, Modena, Italy; ^3^ VIMM, Venetian Institute of Molecular Medicine, Padova, Italy; ^4^ Norwegian School of Sport Sciences, Department of Physical Performance, University of Oslo, Oslo, Norway

**Keywords:** aging, immunosenescence, microarray, blood cells, exercise

## Abstract

The availability of reliable biomarkers of aging is important not only to monitor the effect of interventions and predict the timing of pathologies associated with aging but also to understand the mechanisms and devise appropriate countermeasures. Blood cells provide an easily available tissue and gene expression profiles from whole blood samples appear to mirror disease states and some aspects of the aging process itself. We report here a microarray analysis of whole blood samples from two cohorts of healthy adult and elderly subjects, aged 43±3 and 68±4 years, respectively, to monitor gene expression changes in the initial phase of the senescence process. A number of significant changes were found in the elderly compared to the adult group, including decreased levels of transcripts coding for components of the mitochondrial respiratory chain, which correlate with a parallel decline in the maximum rate of oxygen consumption (VO2_*max*_), as monitored in the same subjects. In addition, blood cells show age-related changes in the expression of several markers of immunosenescence, inflammation and oxidative stress. These findings support the notion that the immune system has a major role in tissue homeostasis and repair, which appears to be impaired since early stages of the aging process.

## INTRODUCTION

The demonstration that some disease states are mirrored by gene expression profiles in blood cells has stimulated the analyses of gene expression changes in blood cells in different physiological and pathological conditions, including aging [1, 2]. A number of studies focused on candidate genes, while others used a genome-wide approach, such as microarray analyses, that provide an unbiased way to investigate the expression of the whole transcriptome. Age-related changes have been the object of a limited number of microarray analyses, however the different designs of available studies does not allow one to draw definitive conclusions about the existence of a specific “aging-signature” in blood cells. We report here a study on the effect of aging in whole blood cells in a selected cohort of healthy subjects from two specific age groups, taking advantage of different bioinformatics approaches for data analysis.

The scope and objectives of this work differ from those of previous studies. For example, one study involved two heterogeneous cohorts, including both males and females and spanning a wide age range (23 to 77), and identified only 16 genes showing a significant positive or negative correlation with age [3]. Another study was specifically focused on the identification of genes related to increased longevity and thus compared gene expression profiles in blood cells from nonagenarians with their middle-age offspring using the partners of the offspring as population controls [4].

In contrast, we focused on a restricted age segment, by comparing subjects in their mid 40s with those in their mid 60s, in order to identify gene expression changes in this initial period of the senescence process. Our preliminary functional studies revealed that the maximum oxygen uptake (VO2_max_), which reflects aerobic fitness, is significantly decreased in this specific phase of the life cycle, in agreement with previous studies [5, 6]. While microarray analysis provides an unbiased view of gene expression profiles reflecting the whole genome, we were especially interested in two specific types of changes. The first concerns genes coding for mitochondrial energy metabolism, to see whether changes in blood cells mirror those found in other tissues and may thus correlate with the global decrease in maximum oxygen uptake. The second concerns the effect of aging on immune cells and immune function, in relation with the notion of immunosenescence, i.e. the functional deterioration and remodeling of the immune system, which negatively impacts both innate and adaptive immunity [7]. Indeed, aging affects various aspects of innate immunity, with a decline in the function of NK cells [8], neutrophils and monocytes [9, 10], leading to impaired phagocytosis and chemotaxis [3]. Adaptive immunity is also affected, with decreased numbers and proportions of CD4+ and CD8+ naïve T cells [11, 12] and accumulation of late-stage differentiated effector memory CD4+ and CD8+ T cells [13] and of Th17 cells [14]. Aging is also associated with a decline in naïve B cell production, leading to an accumulation of memory B cells and a less robust response to antigen [5].

The interest in immune cell changes in gene expression during aging is further stimulated by new studies supporting the notion that immunological mechanisms are not only essential in the response to pathogenic microbes and tumor cells, but have a wider homeostatic role in tissue repair by affecting stem cell function in different tissues. For example, it has been shown that reduced macrophage amount and changes in monocyte/macrophage polarization play a pivotal role in skeletal muscle regeneration [15]. Aging results in a decline in the number of macrophages present in the skeletal muscle and in a defective regulation of their function [16]. Furthermore, recent studies revealed that regulatory T cells (Tregs) stimulate the regeneration of mouse skeletal muscle by promoting the activation of the muscle stem cells, the so called satellite cells [17], and that the impaired regeneration of mouse skeletal muscle during aging is in part due to the reduced recruitment, proliferation, and retention of Tregs in injured muscle [18]. This finding presumably reflects changes in gene expression between Tregs from young and old mice, with particular reference to genes coding for chemokine receptors [19]. Interestingly, these changes are reversible, as the supplementation with specific interleukins, such as IL-33, induces an increased Treg population and enhances muscle regeneration in injured muscles of old mice [18].

The demonstration that specific immune cell populations have a major role in tissue homeostasis supports the possibility that age-dependent changes in immune function may not only mirror but in fact contribute to the aging process of the whole body, thus providing a further stimulus for in depth study of the immune function during aging. The detailed analysis of age-related changes in blood cell gene expression is a step in this direction.

In order to characterize the genes and pathways specifically involved in the process of immuno-senescence, and to further validate the use of whole blood derived RNA as a kaleidoscope through which one can observe the direct effect of aging on blood cells we recruited two groups of healthy men in the local area of Verona. The first group consisted of adult/middle-aged males around 46 years old and the second group was comprised of elderly males around 68 years old. We chose these two age windows because several aspects related to the decline of physiological functions and metabolic responses are preserved in adults/middle-aged, yet decline in the elderly [11]. Our results show that several of the effects of aging are detectable even from samples obtained from within this narrow and relatively close age window (~46 vs ~68). We observed differentially expressed genes and pathways related to immunosenescence, inflammation and systemic aging. Furthermore, we report that transcriptional signatures of blood cells related to mitochondrial function are positively associated with the aerobic capacity of the subjects.

## RESULTS

### Clinical and anthropometric characteristics of the two groups

Characteristics of the participants in the two groups of healthy individuals are reported in Table 1. The adult and elderly groups consisted of 11 and 9 subjects respectively, with average age of 46 ±3 and 68±4 years. All volunteers were physically active, although not performing specific training programs. In agreement with previous studies the two groups had significantly different values of glucose and C reactive protein (CRP) [20, 21], which were significantly higher in the elderly (Table1). The blood cell profile was also different with an age-related expansion of the neutrophils, eosinophils granulocyte compartment, including both neutrophils, eosinophils and basophils. However, all parameters were still within the normal physiological range.

**Table 1:** Summary of the characteristics of the subjects enrolled in the study

	Adults	Eldelry	p. value
Subjects number	11	9	
Gender	male	male	
Age	46 ± 3	68 ± 4	*
BMI (kg/m2)	25.3 ± 1.9	27 ± 2.6	
sBP (mmHg)	121 ± 11	127 ± 16	
dBP (mmHg)	84 ± 9	81 ± 7	
AST (IU/L)	30 ± 6	28 ± 6	
ALT (IU/L)	29 ± 9.4	23 ± 9	
Glucose (mg/dL)	86 ± 9	102 ± 11	***
Cholesterol (mg/dL)	217 ±36	202 ± 39	
HDL (mg/dL)	56 ± 16	52 ± 8	
Triglycerides (mg/dL)	99 ± 30	93 ± 27	
C reactive protein (CRP)(mg/L)	0.8 ± 0,7	2.5 ± 2.3	**
RBC (104/μL)	506 ± 58	494 ± 44	
Platelet (104/μL)	24.8 ± 4.8	26.7 ± 7.0	
WBC (/μL)	6290 ± 1245	6440 ± 1060	
Neutrophil (/μL)	3296 ± 955	3906 ± 698	*
Eosinophil (/μL)	342 ± 189	214 ± 156	*
Basophil (/μL)	46 ± 16	26 ± 15	*
Monocyte (/μL)	338 ± 86	323 ± 56	
Lymphocyte (/μL)	2141 ± 709	1920 ± 720	
Monocyte (/μL)	338 ± 86	323 ± 56	

Data are expressed as mean ± SD

* Student's T test p. value < 0.05

** p. value < 0.01

*** p. value < 0.001.

### Identification of genes differentially expressed in blood samples from adults and elderly subjects

**Figure 1:**
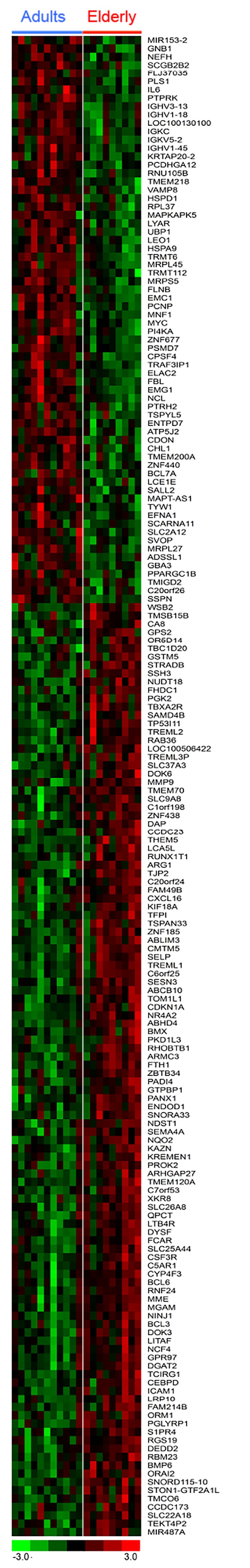
Heatmap of differen-tially expressed genes between blood samples of Adult and Elderly groups Expression profile of 180 genes that differed between Adults and Elderly (p < 0.01). Each gene is represented by a row; each subject by a column. The largest cluster of probes show enhanced expression (transition from green to red), and another cluster show reduced expression (transition from red to green) in the Elderly as compared to Adult. The full set of genes is reported in Table S1.

**Figure 2:**
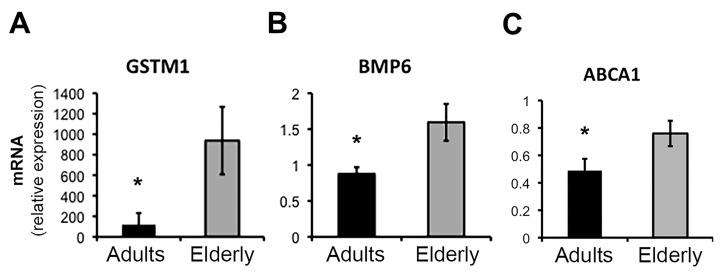
Real-time PCR (qPCR) validation of microarray results for selected genes Quantitative analysis by qPCR of the expression levels of GSTM1 (**A**), BMP6 (**B**), and ABCA1 (**C**) genes in samples from the Adult (n=11) and Elderly (n=9) groups. Data were normalized against GAPDH housekeeping gene. Error bars, SEM. * p < 0.05.

Total RNA was isolated from whole blood samples obtained in the morning, after overnight fasting. Gene expression was determined using whole-transcript arrays (Affymetrix HuGene 1.0 ST) and expression data were analyzed both at gene and pathways level. The elderly group showed increased the expression of 492 genes and decreased the expression of 418 genes (ANOVA p <0.05) (Table S1), by using a more stringent criteria (p < 0.01) the total number of differentially expressed genes was reduced to a more discrete 180 genes (112 upregulated e 68 down-regulated) (Table S1 and Figure 1). Only 4 genes were dysregulated more than 2-fold (GSTM1, CCDC23, TREML3P up-regulated; IGHV1-18 down-regulated) with aging. Changes of the expression levels of a group of representative genes were validated by qPCR (Figure 2A-C). Analysis of the gene list using DAVID [12] showed that the genes up-regulated in the elderly group shows that they are associated with categories such as Immunity and defense, Lipid, Fatty acid and steroid metabolism, Detoxification, Intracellular signaling cascade; whereas the down-regulated genes were associated with Protein biosynthesis and Protein folding (Table S2).

### Age-related alteration of pathways related to immune function

We noted age-related dysregulation of several factors involved in differentiation pathways of immune cells, such as the nuclear receptor NR4A2, the BMX kinase and the two antagonists BMP6 and NOG, which were up- and down-regulated respectively in the elderly group (Figure 1 and 2B).

**Figure 3:**
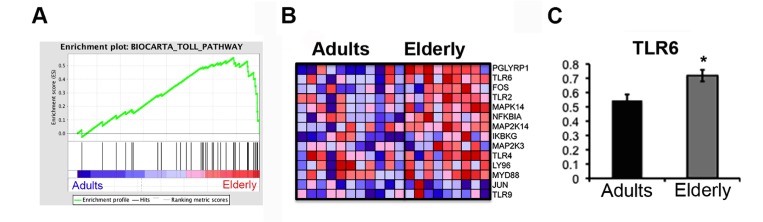
The Toll Pathway gene set is enriched in the Elderly group Gene set enrichment analysis (GSEA) was performed with microarray data. (**A**) Enrichment plots for the Biocarta Toll Pathway gene set are shown. Genes related to the Toll Pathway most strongly associated with the Elderly phenotype are represented on the far right. (**B**) The heatmap shows the relative gene expression (red = high, blue = low) for each gene of the core enrichment for the samples of the two groups (Adults, Elderly). (**C**) qPCR analysis of the expression levels of the TLR6 gene in samples from the Adult (n=11) and Elderly (n=9) groups. Data were normalized against GAPDH housekeeping gene. Error bars, SEM. * p < 0.05.

To gain insight into the biological mechanisms involved in immunosenescence we performed gene set enrichment analysis (GSEA), a computational method that determines whether an apriori defined set of genes shows statistically significant, concordant differences between biological states [13]. This method revealed a significant age-related enrichment (FDR<0.05) of categories related to biological pathways involved in signaling, immunity and cellular metabolism, such as the MET pathway, that has been associated with activated monocytes [14], with the Rho pathway and Toll like receptors (TLRs) signaling among the most over-represented pathways in the elderly group (Table 2). In this gene set we detected three different pattern recognition receptors (TLR4, TLR6, TLR9), the adaptor protein Myd88, the regulatory factors NEMO (IKBKG) and NF-kBIa. The higher expression level of TLR6 was confirmed by quantitative PCR (Figure 3). Using gene sets from the Immunological Signatures database, we noted the related enrichment in the elderly group for genes up-regulated in monocytes following LPS treatment (NES=2.47, FDR<0.0001) (Table 2).

**Table 2:** GSEA analysis of pathways positively and negatively associated with ageing

GROUP	Data Base	Pathway	NES	FDR (<0.05)
**ELDERLY**	*Biocarta*	INTEGRIN PATHWAY	2.17	0.010
		RHO PATHWAY	2.10	0.014
		MET PATHWAY	2.04	0.017
		TOLL PATHWAY	2.03	0.014
		VDR PATHWAY	2.04	0.021
		GRANULOCYTES PATHWAY	1.96	0.028
		PTEN PATHWAY	1.93	0.033
	*Kegg*	ABC TRANSPORTERS	2.11	0.009
	*Immunological Signatures*	LOW LPS VS CTRL TREATED MONOCYTES UP	2.47	<0.0001
		LOW LPS VS VEHICLE TREATED MONOCYTES UP	2.30	<0.0001
**ADULTS**	*Reactome*	TRANSLATION	−2.27	<0.0001
		mRNA SPLICING	−1.84	0.030
		RESPIRATORY ELECTRON TRANSPORT	−1.85	0.026
	*Kegg*	RIBOSOME	−2.18	0.001
		AMINOACYL TRNA BIOSYNTHESIS	−1.77	0.040
		SPLICEOSOME	−1.80	0.058

Gene sets significantly enriched in the Elderly (positive NES) or in the Adult groups (negative NES) are reported. NES, normalized enrichment score; FDR q-val, probability for false discovery rate.

Finally, using as reference the KEGG database we found that the class of ABC transporters, a family of proteins that utilize ATP hydrolysis to transport a wide variety of substrates across various cellular membranes, was enriched in the elderly group. In this gene set ABCC4, ABCA1 and ABCG1 showed the highest level of up-regulation in the elderly, with ABCA1 changes confirmed by qPCR (Figure 2C).

### Aging is accompanied by reduced expression of mitochondrial genes

**Figure 4:**
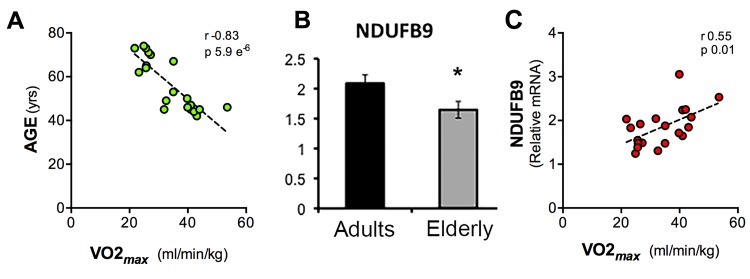
Scatter charts of values of Age, VO_2max_ and NDUFB9 expression levels Graphical representation of the correlation between VO_2max_ and Age (**A**), expression levels of NDUFB9 assessed by qPCR and normalized against GAPDH (**B**), correlation between or VO_2max_ and NDUFB9 gene expression levels detected by qPCR (**C**). (Pearson correlation, *p =* 5.9 e^−6^; *r* = 0.83 (**A**), *p =* 0.01; *r* = 0.55 (**B**), error bars, SEM * p < 0.05).

KEGG and Reactome pathways revealed that many genes coding for subunits of the electron transport chain (ETC) complexes are significantly down-regulated in the elderly group (FDR= 0,026; NES= −1,85) (Table 2). In particular the transcripts of 33 mitochondrial genes of nuclear origin were under-represented in the elderly (Supplementary Figure 1), with reduced levels of the complex I component NDUFB9 also confirmed by qPCR (Figure 4B).

We asked whether these changes observed at the transcriptional levels in blood cells are associated with a global functional parameter of aerobic fitness, such as the maximum rate of oxygen consumption (VO_2*max*_), which declines in the elderly group (Figure 4A). We ran the GSEA using VO_*2max*_ values as a reference parameter and were able to identify a number of pathways positively and negatively associated with VO_*2max*_ (Table S3). Interestingly, a group of gene sets coding for mitochondrial respiratory chain components was positively and significantly associated with VO_2*max*_. For example, NDUFB9 expression levels, as determined by qPCR, were decreased in the elderly group (Figure 4B) and were significantly correlated with VO_2*max*_ (r 0.55, p 0.01) (Figure 4C).

### Additional age-dependent changes in gene expression

According to gene sets from the KEGG and Reactome database, elderly subjects showed reduced content of transcripts associated with protein synthesis (Table 2). In particular, one of the most under-represented gene sets in the elderly group was “Translation” (NES −2.27, FDR <0.0001). In this category we found several transcripts coding for numerous ribosomal proteins and the transcripts of 6 different subunits of the eukaryotic initiation factor 3 (eIF3A, eIF3B, eIF3D, eIF3G, eIF3I and eIF3K).

Transcripts of genes in the management of cellular oxidative stress and detoxification were consistently increased in blood cells of elderly subjects, including GSTM1, GSTT1, GSTM5, NQO2, PRDX5, GPX3 (Figure 2A and Table S1).

Finally in agreement with a recent study investigating age-related changes in gene expression from whole peripheral blood [3], CD248 and SLC4A10, coding respectively for the transmembrane glycoprotein endosialin and for a sodium bicarbonate transporter, were significantly down-regulated in the elderly group (ANOVA p < 0.05; Figure 1 and Table S1).

## DISCUSSION

### Haematological markers of inflammation, immuno-senescence and frailty

Comparative haematological analysis of blood samples from the adult/middle-aged and elderly groups highlight various elements suggestive of systemic inflammation, such as the increased number of neutrophils and CRP levels in the elderly group. These elements have also been proposed as markers for immunosenescence and frailty in comparisons between young and old persons, and in a cohort of 85 year old individuals [22]. Although there is still a debate about whether the number of neutrophils changes with age, recent studies associated an age-related increase in the abundance of neutrophils to low grade inflammation and frailty [23, 24]. Interestingly, when elevated beyond the physiological range, increased neutrophil number is correlated with increased mortality during in the following 2 years, a phenomenon linked to other markers of inflammation such as increased serum levels of IL-6 or CRP [25].

### Age-related changes of factors involved in differentiation and cell stress responses

Our microarray-based comparison of blood transcripts from middle-aged and elderly groups also highlighted various biomarkers implicated in senescence of the immune system, and in the expansion of the granulocyte compartment.

An important finding of this study is the up-regulation of a group of transcripts encoding for proteins with protective roles against oxidative stress, in support of the free-radical theory of aging [26] and the notion that successfully managing oxidative stress can help extend lifespan [27]. In particular, the expression level of GSTM1 has a protective effect, and was associated with reduced mortality in participants of ilSIRENTE study [28], whereas GSTT1 was associated with lower cancer risk, longevity and aging in human granulosa cells [29]. Conversely reduced expression of GSTM1 is associated with prostate cancer and leukemia [30] and its copy number profile is correlated with prognosis [31].

The elderly group also showed dysregulation of factors involved in the differentiation of lymphocytes. In particular, transcripts for nuclear receptor NR4A2 are up-regulated in the elderly group. NR4A2 has been implicated both in the control of differentiation of T helper 17 cells [32], but it is also involved in lipid loading and inflammatory responses in macrophages at atherosclerotic lesions [33].

Our data also suggest a progressive imbalance of the Bmp6/Noggin ratio that may negatively affect proliferation, differentiation and activity of many cell types, and may be related to the pathophysiology of neurodegenerative diseases. Interestingly, there was an inverse relationship between BMP6, which was increased in the elderly group, and Noggin, which was expressed at higher levels in the adult/middle-aged group. BMP6 codes for Bone morphogenetic protein 6, a member of the TGFbeta family that has recently been indicated as an inhibitor of both Ig production and proliferation in memory and naive B cells [34]. BMP6-mediated inhibiton of proliferation can be reversed by treatment with its extracellular natural antagonist Noggin, coded by NOG. Interestingly, BMP6 has been recently associated with Alzheimer disease and reduced neurogenesis both in human patients and in transgenic mice [35].

We also noted an age-related increase of transcripts encoding for the Bone marrow kinase on chromosome X (BMX), a cytosolic kinase expressed in different tissues and in several types of cancer [36]. During the inflammatory response BMX modulates the secretion of pro-inflammatory cytokines induced by TNF, IL-1β, and TLR4. BMX genetic ablation in mutant mice resulted in protection from arthritis [36], suggesting that its up-regulation in the elderly blood cells contribute to the onset of chronic inflammation and may represent a novel therapeutic target in contexts outside of cancer.

### Age-related signaling pathways in blood cells

GSEA analysis highlighted a number of pathways associated with aging. The finding that genes of the Rho signaling pathway are up-regulated in relation with human aging opens the scenario to a wide range of hypotheses and speculations. Rho is a small GTPase that plays a pivotal role in the transduction of mechanical stimuli generated by specific transmembrane proteins (integrins and stretch-activated cell-surface receptors) [37], however in endothelial cells also oxidative stress can also activate the Rho signaling pathway, inducing an increase of arginase I activity [38]. Thus, since oxidative stress and arginase levels are increased during aging, it is possible to conceive that Rho signaling is more active in aged cells and play a relevant role in vascular function/dysfunction [38]. Furthermore changes in extracellular matrix (ECM) stiffness can modulate Rho signaling, and aging is associated with increased endothelial permeability as a function of stiffness of the ECM, and increased Rho activity [39]. However, there is not much data about Rho signaling in hematopoietic cells, although several studies point to RhoA as an important player in the regulation of cell adhesion and differentiation. In particular, RhoA has been implicated in the proliferation and differentiation of pre-T cells, as well as in the migration and activation of T-lymphocytes [40]. It is attractive to speculate that the Rho pathway could also become more active with aging as a response to changes in the microenvironment.

Toll-like receptors (TLRs) are pattern recognition receptors that play a key role in alerting the immune system to the presence of microbial infections. Once activated, the TLRs trigger an inflammatory cascade through the activation of NF-kB-mediated transcription and the consequent production of cytokines and type I interferon, shaping both the innate and adaptive immune responses [41]. TLRs are expressed by monocytes, NK cells, DCs, and B and T lymphocytes [42]. Recent studies indicate that aging influences the function of pattern recognition receptors (PRRs), although results are sometimes controversial, possibly due to different experimental protocols and recruitment criteria [43]. Our data show increased expression levels of TLR6, TLR4 and TLR9. TLR6 and TLR4 recognize lipopeptides and LPS, respectively, although FFAs and elevated glucose levels can also activate TLRs [44]. Changes in serum composition during aging resulting from altered metabolism, glycemia and lipidemia may thus tune systemic inflammation, and eventually contribute to the development of chronic diseases (insulin resistance, atherosclerosis, Alzheimer's disease). TLR4-Myd88 signaling is involved in the development of insulin resistance in high fat diet fed mice [45], reinforcing the notion that lipid-receptors enable innate immunity to sense the lipid and glucose environment and to modulate the inflammatory responses [46]. This is consistent with the enrichment in the elderly group of two immunological signatures for genes up-regulated in monocytes following LPS treatment (Table 2) and with the up-regulation of the BMX kinase, suggesting that monocytes in the elderly group may be in a pro-inflammatory state.

The Met-pathway, also up-regulated in the elderly, is triggered by the pleiotropic cytokine HGF (Hepatocyte Growth Factor) and its tyrosine-kinase receptor c-Met [47]. The HGF receptor is expressed in endothelial cells, erythroid precursors and has also recently been characterized in macrophages [14]. In human monocytes the endotoxin LPS and the pro-inflammatory cytokine IL-1β can induce the expression of both HGF and c-Met. In a sort of autoregulatory loop HGF induces the up-regulation of HGF, its receptor (c-Met) and other pro-inflammatory cytokines [14]. In experimental autoimmune encephalomyelitis (EAE) this pathway promotes proliferation of classically activated macrophages, suggesting a pro-inflammatory role for HGF [48]. These results further suggest that aging favors a pro-inflammatory profile of monocytes and macrophages also through autocrine/paracrine loops mediated also by HGF. It has been suggested that HGF may have a negative effect on muscle regenerative capacity, since high levels of HGF induce satellite cell quiescence by stimulating myostatin expression [49]. Thus sustained systemic levels of HGF may constitute a link between immunosenescence and sarcopenia, although further studies are needed to investigate this relationship.

Finally, the age-dependent increase in the ABC transporters, ABCA1 and ABCG1, is of interest because they mediate active cholesterol efflux toward a variety of exogenous acceptors, including HDL, LDL, liposomes, and cyclodextrin. ABC transporters are particularly relevant for macrophages, since combined deficiency of ABCA1 and ABCG1 promotes foam cells accumulation and accelerates atherosclerosis [50], and also impairs macrophage migration [51]. ABCA1 and ABCG1 transporters thus play a synergistic role in preventing atherosclerosis and cardiovascular disease, making them a useful marker for healthy aging [50].

### Indicators of reduced protein synthesis in aged blood cells

We also report several biological pathways that were down-regulated in the elderly group consisting of sets of genes related to the biosynthetic capacity and metabolism. From the GSEA analysis one of the most depleted gene sets in the elderly is “Translation” (Reactome), including a large number of genes coding for ribosomal proteins and factors involved in the phases of initiation and elongation of translation. Six genes coding for components of the highly conserved large scaffold complex eIF3 were down-regulated, suggesting that binding of the mRNA and the interactions with the 40S ribosomal subunit could become progressively impaired or less efficient with aging. In mammalians eIF3 plays a crucial role in several steps of the initiation of mRNA translation affecting cell growth as well as cancer [52]. The balance between muscle hypertrophy and atrophy is also controlled by eIF3 [53] and one wonders whether the consequent impaired protein synthesis, contributing to the onset and progression of sarcopenia in skeletal muscle, may reflect a more general age-related impairment of the translation process affecting also the blood cells.

### Mitochondrial gene expression

An additional gene set was found down-regulated by aging: the “Electron Transport Chain” (ETC). The idea that mitochondria play a causative role in the progression of aging was first proposed in 1956 with the “free radical theory” by Harman [26], and altered mitochondrial function is a well characterized consequence of aging. The ETC, that drives the transfer of electrons from NADH and FADH2 to molecular oxygen to produce ATP, is composed of 5 complexes organized in supercomplexes. Alterations of these supramolecular engines, by mutations or alteration of proper import and/or folding of the component proteins, or imbalance of stoichiometry, can impair mitochondrial function and contribute to age-related degeneration of several tissues, such as brain, lymphocytes and heart [54]. In this context, our data extend the control phases for the correct assembly of the ETC also to the transcriptional level, showing that a group of nuclear genes encoding for elements of the ETC are generally down-regulated by aging. These observations are in agreement with the demonstration of decreased ETC enzyme activities in aging rat brain and lymphocytes [54], and with age-dependent transcriptome and proteome mitochondrial changes in heart [55] and skeletal muscles [56]. In our samples the complex most severely affected at the transcriptional level is complex *I*. In rats it has been shown that the activities of the various ETC complexes (I-V) undergo a decline with increasing age, and that this decay follows a parallel pattern in brain, lymphocytes and skeletal muscle [54, 57]. Using a multi-disciplinary approach it has been shown that senescence implies the down-regulation of the expression levels of transcripts correlated with reduced activity of ETC complexes in myocardium mitochondria [55]. Aging causes a decay of whole body aerobic function, as determined by maximal oxygen consumption and in humans this decline is accelerated after 60 years of age. The age-related decline in VO_2*max*_, at whole body level, is the results of adaptations occurring both at central and peripheral level, including the reduction in maximal heart rate and lean body mass [6]. Although we cannot take in the relative contribution of each system, mitochondrial dysfunction in different tissues contributes to the age-associated reduction of VO_*2max*_ and it is of interest that this dysfunction is reflected in the decreased levels of transcripts of respiratory complexes in blood cells.

### Comparison with previous studies

In this study we chose to perform microarray analysis on whole blood samples to avoid alteration in gene expression during the separation of the various cell types [58]. Two previous studies have reported gene expression profiling of whole blood cells in relation with age. Nakamura and colleagues analyzed whole blood RNA [3] in a large cohort of subjects, both male and female, of age between 23 and 77. They found 16 genes strongly correlated with aging (11 up-regulated and 5 downregulated). Of these SLC4A10 and CD248 showed the strongest correlation with age. Our dataset confirms this result, since CD248 and SLC4A10 were both significantly down-regulated in the elderly group. CD248 encodes for the transmembrane glycoprotein endosialin, which has been associated to stromal cells in multiple types of cancer but its expression in human blood cells is restricted to naïve CD8+ T-cells, in which it acts as an inhibitor of cellular proliferation maintaining naive T-cells in a quiescent state [59]. Its lower expression in the elderly would indicate a lower abundance of naive T-cells in this population, a well-characterized feature of immunosenescence.

Another study had a more specific objective, namely to identify genes associated with familial longevity [4]. In this study blood samples were obtained from participants of the Leiden Longevity Study, comprising nonagerian sibling pairs, their middle-aged offspring and the partners of the offspring as population controls. A number of genes that represent a potential “longevity-signature” were thus identified [4]. In that report the pathway most closely associated with longevity was the Rho signaling pathway, associated to cellular growth and cytoskeletal organization, and by its connection with the mTOR pathway has been linked to lifespan and health. One of the major limitations of this study is the limited size of the subjects involved, however other studies in which highthroughput technologies combined to physical exercise/inactivity interventions were applied used more limited samples [60-63].

In our study we focused on a more restricted age difference (46 vs 68 years) and used exclusively male subjects. Our results support the notion that crucial changes in gene expression take place in blood cells in this relatively limited time period. Interestingly, some of these changes correlate with global functional changes, such as VO_*2max*_.

## CONCLUSIONS

This study shows that many changes in different pathways and genes occur in a relatively restricted age window, corresponding to the early phase of senescence. Interestingly, similar age-dependent changes in gene expression were also found in many other tissues. As such, blood can serve as a relatively non-invasive surrogate for biomarker discovery. While further studies with larger sample size are required to support this conclusion, our data are consistent with the notion that blood cells can provide a key to understanding systemic events.

## METHODS

### Protection of human subjects

Human subjects research was conducted accordingly to the principles of the Declaration of Helsinki. The study described in this work were in compliance with protocols approved by Institutional Review Board of the University of Verona. All subjects gave their informed consent, and biological specimens and all data collected were anonymized.

### Subjects enrollment, health data collection

For this study twenty healthy men between the ages of 45-55 (n=11) and 65-75 (n=9) years old were recruited in the local area of Verona. They completed a medical-history questionnaire. Exclusion criteria considered are: cardiovascular, respiratory, neurological, endocrine and inflammatory diseases, diabetes or other metabolic disorders, medications affecting the cardiovascular function. Participant characteristics are presented in Table 1.

### Blood sampling

The subjects were asked to avoid vigorous physical activity in the 24 h before blood sampling and to fasten from the evening meal until the morning, when samples were obtained.

Blood was sampled from antecubital vein of each subject while seated in two different occasions, which were separated by at least 7 days. To preserve RNA quality and integrity 3 ml of blood have been collected intoTEMPUS Blood RNA tubes (ABI, Foster City, CA, USA) containing 6 ml Applied BioSystems RNA stabilization reagent.

### Haematological testing

All the samples were processed for routine hematological testing immediately after collection (<15min) on the same Advia 2120i hematology system (Siemens Healthcare Diagnostics, Deerfield, IL, USA) using standard local procedures at GB Rossi Hospital, Verona, Italy.

The parameters tested included red blood cells count (RBC), haematocrit (HCT), haemoglobin (HGB), mean red cell volume (MCV), mean red cell haemoglobin content (MCHC), red blood cell distribution width (RDW), white blood cells (WBC) count, and WBC differential, including lymphocytes, monocytes, neutrophils, eosinophils, basophils and large unstained cells, platelet count, mean platelet volume. The instrument was calibrated against appropriate proprietary reference standard material and verified with the use of proprietary controls.

### Clinical chemistry and immunochemistry test

The clinical chemistry and immunochemistry tests were performed on serum aliquots on the same instrument Cobas® 6000 < c501 > and < e601 > module (Roche Diagnostics GmbH, Penzberg, Germany), according to the manufacturer's specifications and using proprietary reagents. The panel of tests included the following: glucose, total cholesterol, HDL cholesterol, triglycerides, total protein, albumin, urea, creatinine, uric acid, alkaline phosphatase, amylase, aspartate aminotransferase (AST), alanine aminotransferase (ALT), C-reactive protein (CRP). The instrument was calibrated against appropriate proprietary reference standard materials and verified with the use of proprietary quality controls.

### Blood pressure

Blood pressure at rest in sitting position was measured before the evaluation of VO_2*max*_ in the brachial artery by means of a blood pressure monitor (Tango+, SunTech Medical, USA).

### Total RNA preparation and RNA pooling

Total RNA was isolated from each of the two blood samples using Tempus Spin RNA Isolation Kit (Applied Biosystems) as specified in the manufacturer's guidelines. Quality of the purified RNA from was verified on an Agilent® 2100 Bioanalyzer (Agilent Technologies, CA); RNA concentrations were determined using a NanoDrop® ND-1000 spectrophotometer (NanoDrop Technologies, DE). To improve the detection sensitivity of transcripts on microarrays globin mRNA was depleted from a portion of each total RNA sample isolated from TEMPUS Blood RNA tubes using the GLOBINclear™-Human kit (Ambion, TX). To reduce variations due to confounding events, such as spontaneous up- and down-regulation of genes, the two samples of clean RNA from each subject were pooled into one single sample (1ug+1ug RNA).

### cRNA preparation, hybridization, staining, and scanning

The cRNA labeling and hybridizations were performed according to protocols from Affymetrix Inc. (Santa Clara, CA). Briefly, 100 ng of total RNA from whole blood and Globin depleted samples was converted to cRNA and then to sense-strand biotin-dUTP-labeled cDNA using Ambion WT Expression Kit according to the manufacturer's recommended protocols. The single stranded cDNA was then fragmented to ~40-70 nt size by incubating in fragmentation buffer. Fragmented cDNA was assessed for relative length on Agilent 2100 Bioanalyzer and hybridized to Affymetrix Human Gene ST 1.0 chips for 16 hours, washed, stained on an Affymetrix fluidics station and scanned using Affymetrix GeneChip scanner 3000.

### Mircoarray data analysis

Microarray probe fluorescence signals were converted to expression values using robust multiarray average procedure RMA [64] of Bioconductor affy package. Specifically, fluorescence intensities have been background adjusted, normalized using quantile normalization, and expression values for a total of 19 684 custom probe sets calculated using median polish summarization and custom chip definition files for Human Gene 1.0 ST arrays based on Entrez genes (hugene10st_Hs_ENTREZG version 15.1.0) [65]. Quality control was performed using ArrayQualityMetrics [66], and based on the quality assessment all 20 samples were deemed suitable for further analysis.

All data analyses were performed in R version 3.1.2 using Bioconductor libraries of BioC 2.3 and R statistical packages. Raw data are available at Gene Expression Omnibus (GEO) GSE67220.

Genes with statistically significant differential expression between adults and elderly subjects were identified using two-way analysis of variance (ANOVA, p<0.05).

### Quantitative real time PCR

For quantitative Real Time-PCR assays, total RNA was characterized by electrophoresis (Agilent 2100 Bioanalyzer, CA). 400 ng of RNA was converted to cDNA using random primers and Superscript III (Invitrogen, CA). Amplification was carried out in triplicates with an 7900 HT Fast Real Time PCR System (Applied Biosystems) using SYBR green chemistry (Fast SYBR green master mix Applied Biosystems) and a standard 2-step protocol. The primers specific for each gene were designed and analyzed with Primer3 (freeware) and Vector NTI (Invitrogen, freeware). Identity of the amplicons was confirmed by their dissociation profiles and gel analysis.

Quantitative PCR standard curves were constructed by using serial dilutions of muscle cDNAs, using at least 4 dilution points and the efficiency of all primer sets was between 1,8 and 2,2. The data were normalized against *Gapdh* housekeeping gene. Primers are listed in Table S3.

### Evaluation of VO_2max_

All the tests were performed at approximately the same time of the day (22–25°C, 55–65% relative humidity). The subjects were asked to avoid vigorous physical activity in the 24 h before the test and to avoid food intake in the 8 hours before reaching the laboratory. Here they were given a standardized light meal 30 min before starting the test. VO_2max_ and ventilatory thresholds were evaluated during a ramp test performed on an electronically braked cycle ergometer (Excalibur Sport, Lode, Groningen, The Netherlands). The subjects were familiarised with the tasks and asked to perform a maximal incremental ramp test to determine the VO_2max_ [67]. The cycle ergometer seat and handlebar positions were customised for each subject. The ergometer was operated by a metabolic cart (Quark b^2^, Cosmed, Rome, Italy) that also allowed continuous, breath-by-breath measures of gas exchange and ventilation (at the mouth) and HR. The ramp test consisted of 3 min at rest, 5 min of warm-up exercise at 50 W, followed by a continuous increase in the workload by 15-20 W per minute until voluntary exhaustion. The accepted criteria for maximal effort were: respiratory exchange ratio >1.1 and heart rate (HR) >90% of the predicted maximum based on age.

### Over-representation analysis

Over-representation analysis was performed using the Gene Set Enrichment Analysis (GSEA) software (http://www.broadinstitute.org/gsea/msigdg/gsea/msigd). GSEA was applied on linear expression data of the entire dataset. To determine which set of genes shows statistically significant, concordant differences between elderly and adult subjects, we performed GSEA using Signal2Noise as metric and 1,000 permutations of gene set. Gene sets were defined as significantly enriched if the False Discovery Rate (FDR) was < 5%.

To determine VO_2max_ –max ermine VOs < 5%. 5 VO_2max_ value of each subject was used as continuous phenotype labels, and the Pearson's correlation as the metric to select gene sets with expression patterns resembling those encoded in the phenotype labels. Finally, in this case, gene sets were defined as significantly enriched if the False Discovery Rate (FDR) was <5.

### Statistical analysis

All data are reported as the mean ± SEM or SD as indicated in the legend. Statistical analyses between the two groups were performed with unpaired Student's t-test. A P value of < 0.05 was considered significant.

## SUPPLEMENTARY DATA TABLES AND FIGURE




