# Supplementary material for: Aging: a portrait from gene expression profile in blood cells
Source: Aging (Albany NY). 2016 Aug 19;8(8):1802–17. doi: 10.18632/aging.101016 (PMC5032697; doi:10.18632/aging.101016)
Supplement: Supplementary file 1 [file aging-08-1802-s001.pdf]

SUPPLEMENTARY DATA

Data of Supplementary Table S1 is presented in Full Text version of this manuscript.

Table S1. Genes that differed between Elderly - negative Fold Change (FC), and Adults - positive FC ($p < 0.05$).

Supplementary Table S2. Classification of differentially expressed genes (p-value <0.05) according to Panther Biological Processes in DAVID

Biological Process	Genes counted	p.value[a]
Eldelry		
Immunity and defense	58	8.7E-06
Hematopoiesis	7	9.0E-03
Lipid, fatty acid and steroid metabolism	30	1.1E-02
Blood clotting	7	2.6E-02
Detoxification	6	2.8E-02
B-cell- and antibody-mediated immunity	7	3.0E-02
Protein phosphorylation	25	3.1E-02
Lipid metabolism	9	3.3E-02
Intracellular signaling cascade	31	4.1E-02
Adults		
Protein biosynthesis	18	3.4E-03
Protein folding	9	1.3E-02
Hearing	4	2.0E-02
Pre-mRNA processing	11	3.2E-02
Ectoderm development	21	3.4E-02

[a] by Fisher's exact test.

Supplementary Table S3. GSEA analysis of pathways positively and negatively associated with VO_{2max}

NAME	Category	NES	FDR (q-val <0.05)
VO2max positively correlated			
BIOCARTA	TCRA PATHWAY	2.01	0.017
REACTOME	TRANSLATION	2.31	0.001
	SRP DEPENDENT COTRANSLATIONAL PROTEIN TARGETING TO MEMBRANE	2.25	0.001
	3 UTR MEDIATED TRANSLATIONAL REGULATION	2.17	0.002
	RESPIRATORY ELECTRON TRANSPORT ATP SYNTHESIS BY CHEMIOSMOTIC COUPLING AND HEAT PRODUCTION BY UNCOUPLING PROTEINS	2.08	0.004
	RESPIRATORY ELECTRON TRANSPORT	2.06	0.004
	INFLUENZA VIRAL RNA TRANSCRIPTION AND REPLICATION	2.03	0.006
	PEPTIDE CHAIN ELONGATION	2.02	0.006
	INFLUENZA LIFE CYCLE	2.00	0.006
	PURINE RIBONUCLEOSIDE MONOPHOSPHATE BIOSYNTHESIS	1.89	0.031
	TRANSLOCATION OF ZAP 70 TO IMMUNOLOGICAL SYNAPSE	1.86	0.036
	FORMATION OF THE TERNARY COMPLEX AND SUBSEQUENTLY THE 43S COMPLEX	1.86	0.033
	NONSENSE MEDIATED DECAY ENHANCED BY THE EXON JUNCTION COMPLEX	1.84	0.038
	SYNTHESIS SECRETION AND INACTIVATION OF GLP1	1.84	0.036
	ACTIVATION OF THE MRNA UPON BINDING OF THE CAP BINDING COMPLEX AND EIFS AND SUBSEQUENT BINDING TO 43S	1.83	0.037
	RNA POL III TRANSCRIPTION INITIATION FROM TYPE 2 PROMOTER	1.82	0.040
	TRNA AMINOACYLATION	1.80	0.046
	MITOCHONDRIAL TRNA AMINOACYLATION	1.80	0.046
	ACETYLCHOLINE NEUROTRANSMITTER RELEASE CYCLE	1.80	0.044

Supplementary Table S3continue

VO2max negatively correlated			
BIOCARTA	VDR PATHWAY	-2.16	0.018
	UCALPAIN PATHWAY	-2.00	0.025
	RHO PATHWAY	-2.01	0.031
	INTEGRIN PATHWAY	-2.06	0.034
	AHSP PATHWAY	-1.93	0.036
	CELL2CELL PATHWAY	-2.01	0.038
	MET PATHWAY	-1.94	0.038
REACTOME	RESPONSE TO ELEVATED PLATELET CYTOSOLIC CA2	-2.25	0.008
	PLATELET ACTIVATION SIGNALING AND AGGREGATION	-2.15	0.014
	TOLL RECEPTOR CASCADES	-2.13	0.013
	SPRY REGULATION OF FGF SIGNALING	-2.10	0.015
	INFLAMMASOMES	-2.06	0.019
	SMOOTH MUSCLE CONTRACTION	-2.04	0.020
	EGFR DOWNREGULATION	-2.03	0.020
	MYD88 MAL CASCADE INITIATED ON PLASMA MEMBRANE	-2.02	0.020
	ADVANCED GLYCOSYLATION ENDPRODUCT RECEPTOR SIGNALING	-2.02	0.018
	HEMOSTASIS	-2.01	0.017
	SIGNALING BY SCF KIT	-1.96	0.026
	IRON UPTAKE AND TRANSPORT	-1.91	0.044
	ACTIVATED TLR4 SIGNALLING	-1.89	0.046
	TRAF6 MEDIATED INDUCTION OF NFKB AND MAP KINASES UPON TLR7 8 OR 9 ACTIVATION	-1.89	0.044

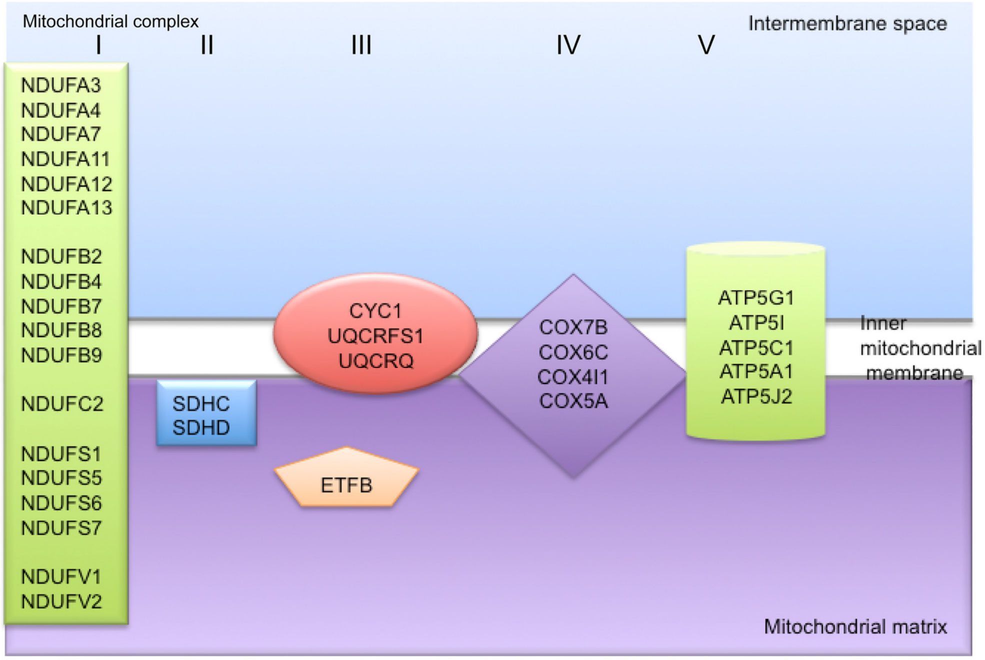

Supplementary Figure S1. Schematic representation of gene coding for subunits of mitochondrial oxidative phosphorylation complexes that resulted under-represented in the blood samples from the Elderly group.
