# Supplementary material for: Aging: a portrait from gene expression profile in blood cells
Source: Aging (Albany NY). 2016 Aug 19;8(8):1802–17. doi: 10.18632/aging.101016 (PMC5032697; doi:10.18632/aging.101016)
Supplement: Supplementary file 2 [file aging-08-1802-s002.docx]

|  | **Adults** | **Eldelry** | **p. value** |
| --- | --- | --- | --- |
| Subjects number | 11 | 9 |  |
| Gender | male | male |  |
| Age | 46 ± 3 | 68 ± 4 | * |
| BMI (kg/m2) | 25.3 ± 1.9 | 27 ± 2.6 |  |
| sBP (mmHg) | 121 ± 11 | 127 ± 16 |  |
| dBP (mmHg) | 84 ± 9 | 81 ± 7 |  |
| AST (IU/L) | 30 ± 6 | 28 ± 6 |  |
| ALT (IU/L) | 29 ± 9.4 | 23 ± 9 |  |
| Glucose (mg/dL) | 86 ± 9 | 102 ± 11 | *** |
| Cholesterol (mg/dL) | 217 ±36 | 202 ± 39 |  |
| HDL (mg/dL) | 56 ± 16 | 52 ± 8 |  |
| Triglycerides (mg/dL) | 99 ± 30 | 93 ± 27 |  |
| C reactive protein (CRP)(mg/L) | 0.8 ± 0,7 | 2.5 ± 2.3 | ** |
| RBC (104/μL) | 506 ± 58 | 494 ± 44 |  |
| Platelet (104/μL) | 24.8 ± 4.8 | 26.7 ± 7.0 |  |
| WBC (/μL) | 6290 ± 1245 | 6440 ± 1060 |  |
| Neutrophil (/μL) | 3296 ± 955 | 3906 ± 698 | * |
| Eosinophil (/μL) | 342 ± 189 | 214 ± 156 | * |
| Basophil (/μL) | 46 ± 16 | 26 ± 15 | * |
| Monocyte (/μL) | 338 ± 86 | 323 ± 56 |  |
| Lymphocyte (/μL) | 2141 ± 709 | 1920 ± 720 |  |
| Monocyte (/μL) | 338 ± 86 | 323 ± 56 |  |
